# Mitochondrial dynamics and metabolism across skin cells: implications for skin homeostasis and aging

**DOI:** 10.3389/fphys.2023.1284410

**Published:** 2023-11-15

**Authors:** Ines Martic, Federica Papaccio, Barbara Bellei, Maria Cavinato

**Affiliations:** ^1^ Institute for Biochemical Aging Research, University of Innsbruck, Innsbruck, Austria; ^2^ Center for Molecular Biosciences Innsbruck (CMBI), Innsbruck, Austria; ^3^ Laboratory of Cutaneous Physiopathology and Integrated Center for Metabolomics Research, San Gallicano Dermatological Institute, Istituti di Ricovero e Cura a Carattere Scientifico (IRCCS), Rome, Italy

**Keywords:** skin, aging, mitochondria, skin homeostasis, skin cells

## Abstract

Aging of human skin is a complex process leading to a decline in homeostasis and regenerative potential of this tissue. Mitochondria are important cell organelles that have a crucial role in several cellular mechanisms such as energy production and free radical maintenance. However, mitochondrial metabolism as well as processes of mitochondrial dynamics, biogenesis, and degradation varies considerably among the different types of cells that populate the skin. Disturbed mitochondrial function is known to promote aging and inflammation of the skin, leading to impairment of physiological skin function and the onset of skin pathologies. In this review, we discuss the essential role of mitochondria in different skin cell types and how impairment of mitochondrial morphology, physiology, and metabolism in each of these cellular compartments of the skin contributes to the process of skin aging.

## 1 Introduction

The human skin is a complex organ that acts as the main barrier of the body against environmental insults (Zhang and Michniak-Kohn, 2012; Panich et al., 2016; Hofmann et al., 2023) and maintains body homeostasis through the regulation of different processes such as water loss, body temperature, and immune response (Chambers and Vukmanovic-Stejic, 2020; Russell-Goldman and Murphy, 2020). Skin aging is caused by the combination of intrinsic and extrinsic factors and leads to a progressive loss of skin function, attenuation of immune efficiency, and increased susceptibility to infections and diseases (Russell-Goldman and Murphy, 2020). The process of intrinsic aging, also known as chronological aging, occurs not just in the skin but in all tissues. This process is mainly related to the passage of time and is influenced by the genetic background and other internal physiological factors. Macroscopically, intrinsic aging of the skin is characterized by thinning of the epidermis, the appearance of fine wrinkles, dryness, loss of elasticity, and uneven pigmentation (Cavinato, 2020). At the molecular level, intrinsic aging is characterized by a complex interplay of factors, where the emergence and accumulation of senescent cells can both result from and coincide with increased oxidative stress, telomere shortening, and damage to organelles and macromolecules (López-Otín et al., 2013; 2023). In contrast, extrinsic aging is induced by environmental insults, such as ultraviolet (UV), cigarette smoke, and air pollution, and is estimated to contribute to the major visible signs of skin aging (Cavinato and Jansen-Dürr, 2017; Wang et al., 2020; Zhang et al., 2023). The characteristics of environmentally exposed skin often overlap with those of intrinsically aged skin. Extrinsically aged skin is characterized by deeper wrinkles, rough texture, and leathery appearance, exacerbated uneven pigmentation, and may be associated with the development precancerous lesions (Cavinato, 2020). Molecular mechanisms involved in extrinsic skin aging are associated with accumulation of DNA damage, increased generation of reactive oxygen species (ROS), telomere shortening, inflammatory processes, and altered cellular signaling pathways, among others (Russell-Goldman and Murphy, 2020). Additionally, appearance and accumulation of senescent cells in the epidermis and dermis is considered one of the hallmarks of skin aging (Ressler et al., 2006; Yoon et al., 2018).

Mitochondria are dynamic organelles enclosed by two membranes that are present in eukaryotic cells. They have their distinct DNA and are essential for meeting the energy and metabolic needs of cells. The primary function of mitochondria is to generate adenosine triphosphate (ATP) via cellular respiration, thereby acting as the main energy source of the cells. Beyond energy production, mitochondria play pivotal roles in the regulation of cell viability, proliferation, calcium homeostasis, and programmed cell death (Vasileiou et al., 2019; Lee et al., 2022). Furthermore, mitochondria are involved in maintaining electrolyte homeostasis, initiating inflammatory responses, and orchestrating cellular redox reactions, particularly by ROS (Lin et al., 2023).

While the research on the role of impaired mitochondrial function on aging has extensively focused on fibroblasts and keratinocytes, the investigation of mitochondrial dysfunction in other skin-residing cell types, including melanocytes, immune cells, sebocytes, and adipocytes, remains largely unexplored (Oblong et al., 2020; Wlaschek et al., 2021; Sturm et al., 2022; Hofmann et al., 2023). In the following sections, we summarize the latest findings about mitochondrial metabolism and physiology in the different compartments of the skin with their residing skin cell types and how the impairment of this organelle in each of these cells contributes to skin aging.

## 2 Overview of skin structure and residing skin cell types

**FIGURE 1:**
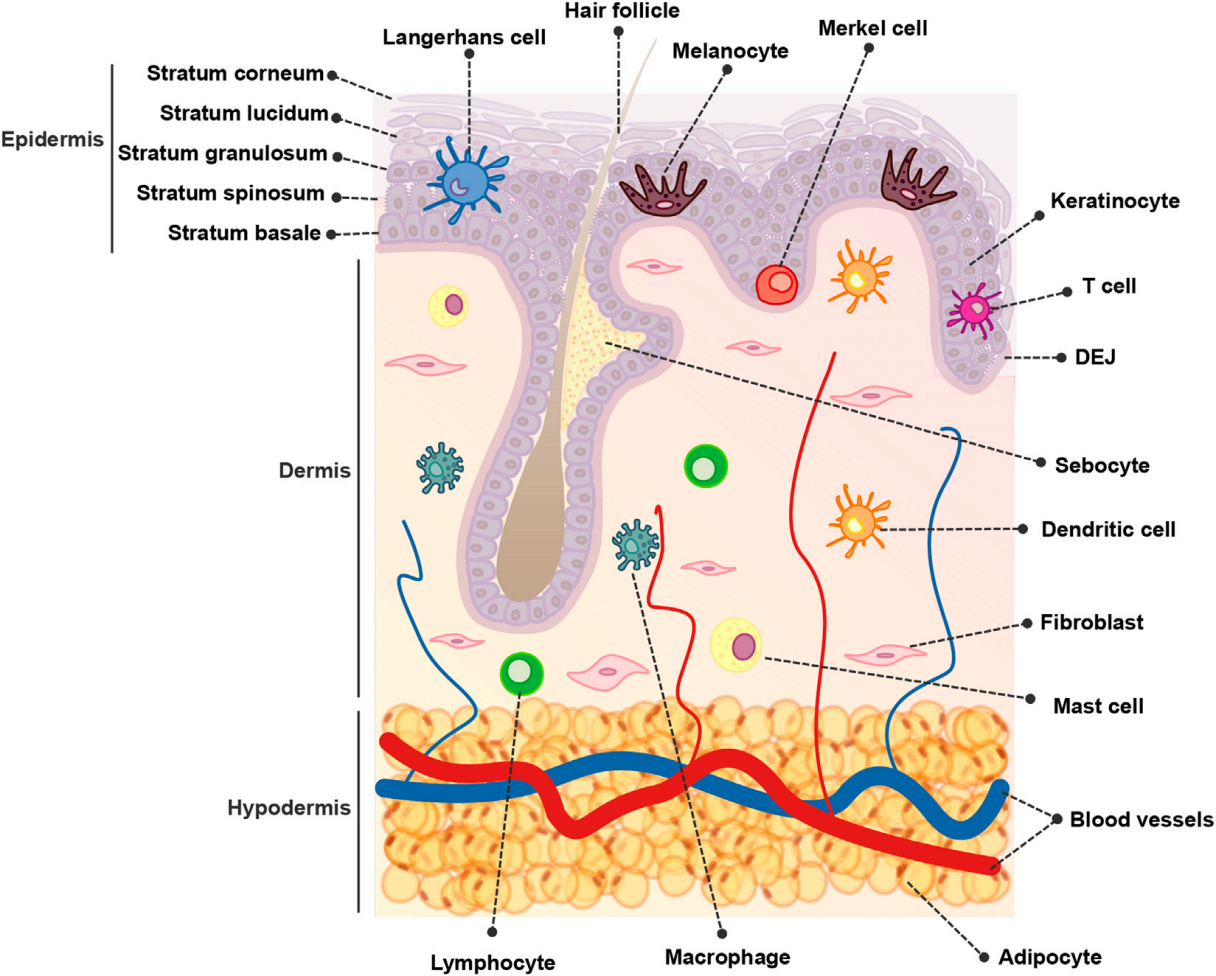
Overview of skin structure and cellular composition.

The skin is composed of over 20 different cell types and is organized into three main compartments: the epidermis, dermis, and hypodermis (Figure 1). The epidermis constitutes the outermost layer, providing a protective barrier to the skin tissue The dermis lies below the epidermis and contains various structures such as blood vessels, hair follicles, and sweat glands. Finally, the hypodermis, also known as the subcutaneous tissue, is situated beneath the dermis and consists of adipose tissue that acts as an insulating layer (Hofmann et al., 2023).

Keratinocytes, derived from the ectodermal layer during embryogenesis, constitute the predominant cell type within the epidermis, forming the protective barrier of the skin. Keratinocytes differentiate as they move from the basal layer towards the skin surface, undergoing structural changes and producing keratins, fibrous proteins that contribute to skin strength and waterproofing (Zhang and Michniak-Kohn, 2012; Hill et al., 2015).

Melanocytes are specialized cells that originate from the neural crest. They reside in the epidermal basal layer along the dermo-epidermal junction (DEJ) and within hair follicles (Lin and Fisher, 2007; Duval et al., 2014). Melanocytes are responsible for the production of melanosomes containing melanin, in a process known as melanogenesis (Cichorek et al., 2013). These melanosomes remain within the melanocytes or are transferred to the neighboring keratinocytes, where melanin is positioned above the nuclei to safeguard their genetic material (Yamaguchi et al., 2007; Mello et al., 2016).

The dermis is connected to the epidermis by the DEJ and is composed of an abundant extracellular matrix (ECM) along with different functional structures and cell types. Within the skin compartments, the dermis hosts the highest cell diversity. Fibroblasts, the major cell type of the dermis, play a pivotal role in the synthesis and maintenance of the ECM, which consists of structurally relevant proteins such as collagen and elastin, glycoproteins, and glycosaminoglycans. Collectively, these elements contribute to the skin’s elasticity and strength (Zhang and Michniak-Kohn, 2012; Wang et al., 2020; Kim et al., 2022; Hofmann et al., 2023). Other cells present in the dermis are mesenchymal stem cells (MSCs) and adipocytes, and due to the presence of blood vessels, sparse immune cell populations (Abdallah et al., 2017). Furthermore, the dermal compartment contains additional functional complex structures such as hair follicles, sebaceous glands, sweat glands, and nerve endings (Hofmann et al., 2023).

The hypodermis, also known as subcutaneous adipose tissue, is the bottom layer of the cutaneous tissue. It is predominantly composed of adipocytes, pre-adipocytes, and adipose-derived stem cells but also of fibroblasts, pericytes, macrophages, T-cells, and erythrocytes in a stromal vascular cell network organized within nerves, muscles, and hair follicles (Trevor et al., 2020). The primary function of the hypodermis is to connect the skin to underlying structures such as muscle and bones, providing support and insulation to the body and serving as a long-term energy storage depot (Mancuso and Bouchard, 2019).

The skin immune system comprises a network of effector cells and molecular mediators. The skin immune system constitutes a highly sophisticated system that provides physical, chemical, and microbiological barriers, to protect the host from external insults and pathogens and helps maintain skin homeostasis (Abdallah et al., 2017; Nguyen and Soulika, 2019).

The primary structure of the skin and the cellular components that inhabit this tissue are depicted in Figure 1.

## 3 Overview of mitochondrial function and the role of mitochondria in aging

**FIGURE 2:**
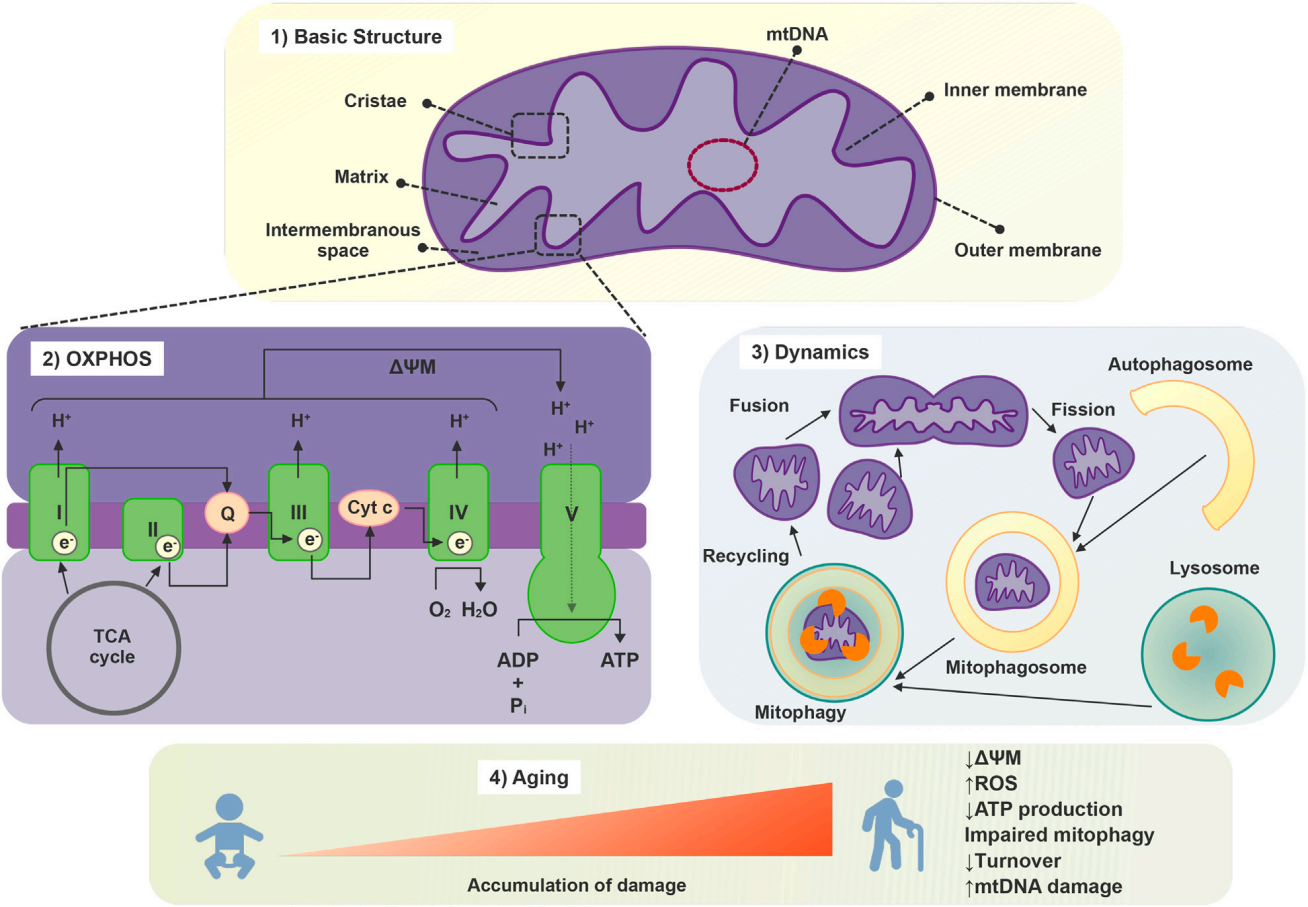
Mitochondrial structure, function, and aging-related changes.

Mitochondria are enclosed by two membranes, namely, the inner and outer mitochondrial membranes. This arrangement forms two distinct aqueous compartments: the intermembrane space and the mitochondrial matrix, which play a crucial role in supporting physiological functions of this organelle. The outer membrane is highly permeable and is composed of many large protein-based pores along with active transporter proteins, which are collectively responsible for the transit of proteins, nucleotides, ions, and metabolites between the cytosol and the intermembrane space. In contrast, the inner membrane has much more restricted permeability, much like the plasma membrane of a cell. The inner membrane forms numerous folds (cristae), which extend into the interior (or matrix) of the organelle in which the proteins involved in electron transport and ATP synthesis are embedded (Figure 2). The intermembrane space lies between the two membranes, and its composition is very similar to the one of the cytosol. The matrix, enclosed by the inner membrane, contains enzymes, mitochondria DNA (mtDNA), and ribosomes and is important for ATP production. mtDNA is packed in nucleoids which are translated into important mitochondrial proteins, including those that are part of the oxidative phosphorylation complexes (Vasileiou et al., 2019).

Mitochondrial metabolism orchestrates an intricate network of biochemical processes within eukaryotic cells, which collectively drive the generation of energy in the form of ATP. Mitochondria engage in oxidative phosphorylation, where electrons derived from metabolic substrates, such as glucose and fatty acids, pass through a series of protein complexes in the electron transport chain. This creates a proton gradient across the inner mitochondrial membrane, which, in turn, powers the ATP synthase enzyme to synthesize ATP (Figure 2). Additionally, mitochondria partake in the citric acid cycle (TCA), or Krebs cycle, which oxidizes acetyl-CoA molecules derived from various nutrients, releasing electrons that feed into the electron transport chain. Furthermore, mitochondrial metabolism encompasses fatty acid oxidation, amino acid catabolism, and the urea cycle (McElroy and Chandel, 2019).

Mitochondria also play a pivotal role in the intricate process of apoptosis, a controlled form of cell death essential for developmental processes and for the maintenance of tissue homeostasis. Mitochondria release factors like cytochrome c, which triggers the formation of the apoptosome a multiprotein complex that activates caspase enzymes. This cascade of events leads to the orchestrated breakdown of cellular components, ultimately resulting in cell death (Cao et al., 2022).

The dynamic nature of the mitochondrial network, which is crucial for maintaining cellular and mitochondrial homeostasis, is regulated by mitochondrial biogenesis, trafficking, fusion and fission processes. These events shape mitochondrial morphology, distribution, and function, allowing for optimal cellular energy production, calcium homeostasis, and other essential mitochondrial activities. Furthermore, this dynamic regulation allows for the degradation of dysfunctional mitochondria, the maintenance of the redox status, and the preservation of mitochondrial integrity, including mtDNA content (Shi et al., 2018; Liu et al., 2020). Mitochondrial biogenesis is a tightly controlled process that involves various signaling pathways and transcription factors. Mitochondrial biogenesis allows cells to meet increased energy demands in response to different stimuli, to replace degraded mitochondria and is essential for the adaptation of cells to stress (Shi et al., 2018).

Recent experimental data evidences that mitochondria can crosstalk and move beyond cell boundaries in a variety of pathophysiological contexts, challenging the paradigm of intracellular segregation of mitochondria and mtDNA inheritance, opening a new era that is leading to the discovery of a more interconnected, dynamic and plastic nature of mitochondrial biology (Dong et al., 2023). Mitochondria-derived material might be transfer to neighboring cells in the form of cell-free mitochondria or included in extracellular vesicles (Valenti et al., 2021; Zorova et al., 2022). This process not only aids in cellular repair but also contributes to the propagation of vital mitochondrial functions. Besides restoring stressed cells and damaged tissues due to mitochondrial dysfunction, intercellular mitochondrial transfer also occurs under physiological and pathological conditions (Liu et al., 2021). The transfer of active mitochondria from MSCs has been identified as a repair mechanism for rejuvenating damaged skin fibroblasts and addressing pathological inherited conditions, as documented in previous studies (Newell et al., 2018; Paliwal et al., 2018; Mohammadalipour et al., 2020). This mechanism underscores its integral role in MSCs’ regenerative capabilities. Notably, A549 cells lacking mtDNA, referred to as A549 ρ° cells, displayed the ability to restore both mtDNA content and a functional mitochondrial pool when co-cultured with human MSCs or skin fibroblasts (Spees et al., 2006). Furthermore, recent research has proposed mitochondrial transplantation as a potential regenerative therapy for addressing skin aging, particularly in the context of replacing UV-damaged mitochondria (Balcázar et al., 2020; Peñaherrera et al., 2023). While these studies demonstrate the promising potential of mitochondrial transfer in cellular rejuvenation and regenerative therapy, it is important to note that to date, there is no research reporting the transfer of mitochondria between skin cells *in vivo*. The extent to which this mechanism is relevant for maintaining skin homeostasis or is implicated in skin pathological conditions remains an open area of exploration. Further investigations into the *in vivo* relevance of intercellular mitochondrial transfer within the skin will be crucial for a comprehensive understanding of its role in skin health and disease (Figure 2).

To ensure their optimal functioning, cells have evolved an elaborate system of mitochondria quality control. These mechanisms involve processes such as mitochondrial biogenesis, fusion and fission events, mitophagy - the selective degradation of excessive or dysfunctional mitochondria via autophagy-, and the activation of molecular chaperones, which are synergistically regulated and interact with each other (Sedlackova and Korolchuk, 2019) (Figure 2).

Independent to the specific cell type, during aging, mitochondria undergo several well documented alterations in structure and function, which lead to insufficiency of mitochondrial quality control and turnover mechanisms (Zhang et al., 2023). These changes contribute to cellular dysfunction and have been implicated in the aging process itself and in the pathogenesis of age-related diseases.

Aging is associated with changes in mitochondrial morphology, including either hyperfusion or increased fragmentation and loss of mitochondrial connectivity (Liesa and Shirihai, 2013; Sreedhar et al., 2020). Additionally, with aging, mitochondria present a decline in the efficiency of oxidative phosphorylation, leading to reduced ATP production, mitochondrial membrane potential (ΔΨ_M_), and consequent compromised cellular energy metabolism. These alterations are related to the increased production of ROS exhibited by mitochondria during aging, the accumulation of which causes oxidative damage to mitochondrial and cell components contributing to cellular senescence (Gao et al., 2022). Furthermore, mitochondrial turnover is decreased during aging as a reflection of downregulated mitochondrial biogenesis and impaired mechanisms of mitochondrial quality control such as mitophagy (Sreedhar et al., 2020). Decline in both mitophagy and autophagy pathways are linked to aging and a variety of age-related pathologies including neurodegenerative, cardiac and autoimmune diseases, diabetes, hepatic dysfunction, as well as neoplasms (Palikaras et al., 2017). The characteristics of the mitochondrial response to stress depend on the stimuli and the cell type. One notable event is the accumulation of somatic mtDNA mutations, due to internal or external insults, which can impair mitochondrial function and contribute to increased ROS production (Zorov et al., 2019; Cavinato et al., 2021) (Figure 2).

### 3.1 Basic mechanisms of cellular senescence and changes in mitochondria during this process

While it serves as a tumor-suppressive mechanism and contributes to tissue repair and to embryonic development (Campisi, 2001; Muñoz-Espín et al., 2013; Demaria et al., 2014) the progressive accumulation of senescent cells in aging tissues has been linked to various age-related pathologies and the overall aging process (Baker et al., 2011; Huang et al., 2022). Notably, in the skin, senescent cells accumulate in both the dermis and epidermis during aging, affecting skin layers’ functions and contributing to age-associated decline in overall tissue function (Ressler et al., 2006; Wedel et al., 2023).

Cellular senescence is a biological process characterized by irreversible cell cycle arrest and a distinct set of phenotypic changes. Senescent cells exhibit enlarged, flattened morphology, increased production of ROS, lipofuscin granule accumulation, and increased activity of senescence-associated β-galactosidase (SA-β-Gal) enzyme (Fortuna et al., 2021). Additionally, alterations in mitochondrial function and protein quality control mechanisms are commonly observed in senescent cells (Cavinato et al., 2016; Martic et al., 2020; Wedel et al., 2023). Senescent cells release a complex mixture of proinflammatory cytokines, chemokines, growth factors, and matrix-degrading enzymes collectively denominated senescence-associated secretory phenotype (SASP). The SASP is believed to have a profound impact on surrounding tissues during aging. It can trigger chronic inflammation, disrupt tissue structure and function, and promote the development of age-related diseases (Birch and Gil, 2020; Cuollo et al., 2020). Inflammation driven by the SASP, often referred to as “inflammaging,” can contribute to tissue degeneration, impaired regenerative capacity, and increased susceptibility to various age-related conditions, including cancer, neurodegenerative disorders, and metabolic diseases (Fülöp et al., 2019).

Mitochondrial dysfunction and cellular senescence are hallmarks of aging and are intricately interconnected (Miwa et al., 2022). Mitochondrial dysfunction, characterized by a decreased respiratory capacity per mitochondrion and reduced mitochondrial membrane potential, often accompanied by elevated production of ROS, is considered both as a cause and consequence of cellular senescence. This dysfunction plays a pivotal role in multiple feedback loops that induce and sustain the senescent phenotype (Chapman et al., 2019). During cellular senescence, the accumulation of damaged mitochondria is driven by factors such as oxidative stress and impaired mitophagy, which selectively removes dysfunctional mitochondria. As damaged mitochondria persist, they exacerbate mitochondrial dysfunction, leading to decreased cellular bioenergetics and heightened oxidative stress (Ghosh-Choudhary et al., 2021). This interplay between mitochondrial dysfunction and senescence not only contributes to cellular aging but also has systemic effects, impacting tissue and organ function, and thereby playing a significant role in the broader manifestations of aging and age-related diseases in organisms.

Mitochondria of skin cells are prone to accumulate damage, which contributes to the process of skin aging (Wang et al., 2020; Zhang et al., 2023). In the following chapters, we will delineate the peculiar role of mitochondria in different cutaneous cell types and how dysfunction of this organelle contributes to progressive functional decline of the cells and skin aging.

## 4 Mitochondria alterations in different cell types during aging

### 4.1 Mitochondria in keratinocytes’ homeostasis and aging

As keratinocytes progress from basal to suprabasal layers and undergo terminal differentiation, mitochondrial function, and morphology undergo dynamic alterations. Additionally, mitochondrial ROS acts as a signaling molecule, which regulates epidermal differentiation (Hamanaka et al., 2013). Metabolically, mitochondria provide intermediates that support biosynthetic pathways required for keratin production and lipid metabolism, thus dysregulation of mitochondrial function in keratinocytes is crucial for the formation of the skin barrier and skin health (Trompette et al., 2022).

When comparing keratinocytes to slowly proliferating cells, such as fibroblasts, the accumulation of functional defects over time is less evident in keratinocytes and mechanisms other than mitochondrial damage also impact epidermal integrity (Yaar and Gilchrest, 2001; Farage et al., 2013; Khalid et al., 2022). Changes in mitochondrial morphology have been observed during keratinocyte differentiation and are implied in the process of skin aging. In contrast to keratinocytes located in the stratum spinosum, those within the stratum granulosum possess a higher quantity of mitochondria that are organized in a more compact mitochondrial network (Mellem et al., 2017). Apart from a consistently strong inter-individual variation in the number of mitochondria in proliferating keratinocytes, no age-associated differences in the number of mitochondria have been observed comparing cells isolated from young and old subjects *in vitro* (Chevalier et al., 2022), whereas mitochondria connectivity is decreased in keratinocytes from older individuals in comparison to younger ones (Mellem et al., 2017).

The elimination of mitochondria from keratinocytes during the process of differentiation plays a critical role in the maturation and stratification of the epidermis. A recent study demonstrated that in the upper epidermal layers, keratinocytes initiate mitochondrial fragmentation, depolarization, and acidification by upregulating the mitochondrion-tethered autophagy receptor BCL2/adenovirus E1B 19 kDa protein-interacting protein 3-like (BNIP3L/NIX). Depletion of NIX hampered epidermal maturation and disrupted the elimination of mitochondria, whereas the overexpression of NIX accelerated keratinocyte differentiation and led to premature mitochondrial fragmentation (Simpson et al., 2021). Furthermore, the expression of BNIP3, another mitophagy receptor highly expressed in the upper layers of the epidermis, decreases with chronological aging in the human epidermis reinforcing the idea that age-related dysfunction of mitophagy impacts epidermal homeostasis (Chevalier et al., 2022). Thus, the elimination of mitochondria by mitophagy seems to be less efficient in aged keratinocytes accompanied by reduced ATP production, suggesting that improper mitochondria dynamics could be involved in impaired cornification of old skin (Xiaoyun et al., 2017). Additionally, the timing of mitochondria elimination from keratinocytes seems to be a well-defined process since mitochondria are necessary during differentiation to keep up with the high metabolic demand before the terminal differentiation but are undesired in corneocytes lacking an adequate intracellular antioxidant system (Thiele, 2001; Thiele et al., 2001).

Basal keratinocytes primarily rely on glycolysis for energy production and as they differentiate and migrate towards the upper layers of the epidermis, there is an increase in mitochondrial oxidative phosphorylation and ATP production through the TCA cycle and electron transport chain (Hamanaka and Chandel, 2013). Given that mitochondria are primary generators of ROS, oxidative stress predominantly manifests in the epidermis’s deepest layer, particularly in the basal layer’s stem cells where keratinocytes heavily rely on mitochondrial activity (Vidali et al., 2023). ROS produced by the activity of mitochondrial oxidative phosphorylation (OXPHOS) complexes in the skin are responsible for several phenotypic aging symptoms such as hair greying and loss and wrinkling of the skin as demonstrated in studies using mice carrying a loss of function mutation in the DNA polymerase ɣ gene (Singh et al., 2018). In contrast, treatment of skin with antioxidants leads to the reduction of ROS levels and consequently to the blockage of keratinocyte differentiation (Hamanaka et al., 2013) reinforcing the complexity of ROS signaling in keratinocyte function.

Increasing evidence demonstrates that mitochondrial ROS generation promotes epidermal differentiation and hair growth by activating the Notch and β-catenin transcriptional programs (Hamanaka and Chandel, 2013). Bhadhuri et al. reported that an early event in keratinocyte differentiation is the mitochondria localization of myelin protein zero-like 3 and enzyme ferredoxin reductase proteins that, in turn, increase ROS to drive Notch-dependent epidermal differentiation (Bhaduri et al., 2015). Furthermore, in mice, keratinocyte-specific ablation of mitochondrial transcription factor A (TFAM), which is required for the transcription and replication of the mitochondrial genome, impaired epidermal differentiation promoting the maintenance of a basal, proliferative phenotype and consequently resulting in an impaired barrier function and high mortality rate (Hamanaka and Chandel, 2013; Hamanaka et al., 2013). In addition, primary keratinocytes isolated from TFAM-depleted mice failed to differentiate *in vitro* and displayed impaired mitochondrial ATP production and increased ROS production (Hamanaka et al., 2013).

From a functional metabolic perspective, aging is linked to modifications in mitochondrial metabolism within primary keratinocytes, encompassing reduced OXPHOS (Dumas et al., 1995; Tan et al., 2019). The age-dependent rise of ROS in the keratinocytes coupled with the concomitant decline in mitochondria membrane potential prompts a metabolic shift from OXPHOS to anaerobic glycolysis. Glycolysis serves as a compensatory mechanism for membrane potential depolarization and the progressive decrease of cardiolipin levels during life (Dumas et al., 1995). OXPHOS and glycolysis also control epidermal stem cell fate in terms of the balance between stemness maintenance and differentiation (Folmes et al., 2012). A low nicotinamide adenine dinucleotide/Nicotinamide (NAD^+^/NAM) ratio impairs keratinocyte differentiation and is associated with the acquisition of premature keratinocyte senescent phenotype (Tan et al., 2019).

Some specific pathologic conditions impact mitochondria integrity. For instance, in the presence of abnormally high glucose levels, such as in diabetic patients, keratinocytes accumulate intracellular ROS, leading to mitochondrial dysfunction and inflammation, a fact that could partially explain cutaneous complications in diabetic patients (Rizwan et al., 2023). A distinctly glycolytic phenotype has been observed in keratinocytes through skin biopsies from the elderly (Prahl et al., 2008). Coenzyme Q (CoQ), a lipophilic prenylated quinone that acts as an electron shuttle between complex I/II and complex III of the electron transport chain, also serving as a ROS scavenger (Hoppe et al., 1999), exhibits high expression levels in the epidermis compared to the dermis but decreases significantly with age (Ben-Meir et al., 2015). Exogenous supplementation of CoQ counters membrane lipid oxidation and effectively restores mitochondrial metabolism in aged or stressed cells (Prahl et al., 2008; Schniertshauer et al., 2016). Furthermore, studies highlight that topically applied CoQ on photo-aged skin improves aging-related phenotypic traits and reinstates mitochondrial function (Blatt et al., 2005; Puizina-Ivić et al., 2010).

Numerous studies have provided evidence that the accumulation of mtDNA damage is associated with age-related changes in various tissues, specifically in the skin, exposure to UV stress can exacerbate this damage. Indeed, an association between Sun exposure and common mtDNA deletions has been demonstrated in both keratinocytes and fibroblasts (Pang et al., 1994; Birch-Machin et al., 1998; Berneburg et al., 2004). Compared to nuclear DNA, mtDNA is highly susceptible to damage given its limited repair capacity (Wallace and Torroni, 2009). Ultraviolet radiation is known to induce deletions in mtDNA, disrupting the flow of electrons and impeding energy production. Furthermore, UV irradiation causes fragmentation of the mitochondrial network within cells. This disruption in the network’s structure impairs its ability to maintain mtDNA stability. As a result, the fusion of healthy and damaged mitochondria is compromised, preventing the exchange of healthy mtDNA and the elimination of damaged copies. Ultimately, these events lead to cellular dysfunction and premature senescence (Stout and Birch-Machin, 2019; Balcázar et al., 2020).

UV radiation directly affects the epidermis. Cumulative Sun exposure harms keratinocytes leading to functional decline of the skin tissue and compromising physiological skin homeostasis (Brand et al., 2018). Excessive UV exposure leads to necrosis in some epidermal keratinocytes. Loss of membrane integrity of these damaged cells facilitates the release of cellular contents into the surroundings. Nearby healthy keratinocytes take in these released molecules, including double-stranded RNA, which activates toll-like receptor 3 in their endosomes. This sets off a cascade of events that increase inflammation and lipid-related processes (Bernard et al., 2012; Borkowski and Gallo, 2014). UVB irradiation of spontaneously transformed aneuploid immortal keratinocyte cell line cells leads to the release of mitochondria and nuclear-damaged DNA culminating in the activation of cyclic guanosine monophosphate-adenosine monophosphate synthase/protein stimulator of interferon genes DNA sensor and inducing an innate immune response (Li et al., 2021). Furthermore, chronic UV irradiation of spontaneously transformed aneuploid immortal keratinocyte cells leads to a decrease in ΔΨ_M_ along with a reduced mitochondrial mass associated with marked reshaped mitochondria (Kelly and Murphy, 2016; Bellei and Picardo, 2020).

A study on the age-dependency of the mitochondrial network in young and old volunteers revealed that keratinocytes in old skin establish a significantly more fragmented network with smaller and more compact mitochondrial clusters than keratinocytes in young skin (Mellem et al., 2017).

UVB irradiation of keratinocytes results in disrupted mitochondrial dynamics, subsequently triggering apoptosis and inflammation. Following UVB irradiation, Hacat cells exhibit an increase in fission protein dynamin-related protein 1 (Drp1), accompanied by a decrease in fusion proteins mitofusin 1 and 2 (Mfn1/2). This alteration promotes heightened production of ROS and activates the inflammatory pathways, including cGAS-STING and the NACHT, LRR and PYD domains-containing protein 3 (NLRP3) inflammasome. Primary human keratinocytes also undergo dose-dependent mitochondrial fragmentation following UVB irradiation. In these cells, depletion of Drp1 leads to mitochondria hyperfusion and attenuates UVB-induced mitochondrial fragmentation (Li et al., 2023).

**FIGURE 3:**
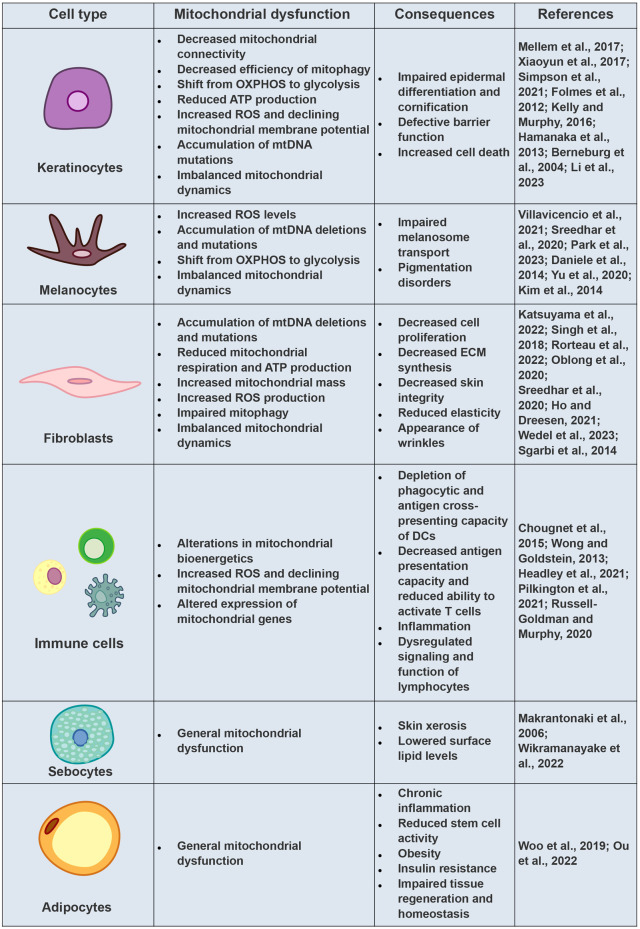
Mitochondrial Dysfunctions and Cellular Consequences in Aging Skin Cells. Comprehensive overview of mitochondrial dysfunctions observed in various skin cell types during the aging process, elucidating their subsequent implications for skin health.

The main mitochondrial damages observed in keratinocytes during aging, along with their resulting consequences for the skin, are displayed in Figure 3.

### 4.2 Mitochondria in melanocytes’ homeostasis and aging

Melanocytes, specialized cells residing in the basal layer of the epidermis, are responsible for producing and distributing the pigment melanin (Cichorek et al., 2013). As the skin ages, the density and function of melanocytes decline leading to several consequences. Firstly, impaired melanin production and distribution can result in uneven skin pigmentation, contributing to the appearance of age spots or hyperpigmentation. Secondly, the diminished ability to protect against harmful UV radiation may increase the risk of sun-induced skin damage, including sunburn and an elevated susceptibility to skin cancers. Lastly, the loss of melanocytes’ regulatory role in immune responses could impair the skin’s defense mechanisms, potentially affecting wound healing and overall skin health (Bellei and Picardo, 2020).

Melanin synthesis is a precisely regulated process that involves various cellular mechanisms and compartments, including mitochondria. A group revealed a novel connection between melanosomes and mitochondria, facilitated by fibril bridges dependent on Mfn2. This unique linkage of melanosome-mitochondria predominantly occurs early during melanosome generation in the perinuclear area. Intriguingly, disruption in the mitochondrial function or impaired melanogenesis correlates with a reduction of interorganelle connectivity suggesting a potential involvement of this mechanism in the development of skin pigmentation disorders (Daniele et al., 2014).

Melanocytes display elevated basal levels of ROS compared to other skin cell types, primarily due to their engagement in the ROS-generating process of melanogenesis (Sreedhar et al., 2020). The role of ROS in pigmentation processes and their contribution to the development of skin pigmentation disorders are the subjects of conflicting findings (Jiménez-Cervantes et al., 2001; Schallreuter et al., 2008; Liu et al., 2009; Cunha et al., 2012; Kim et al., 2013). Generally, melanocytes extracted from sun-exposed skin areas exhibit heightened ROS levels, coupled with increased melanin production, contrasting with melanocytes from sun-protected skin (Sreedhar et al., 2020).

Mitochondrial dynamics play a peculiar role in melanocytes (Yu et al., 2020). It has been demonstrated that in this cell type genetic and chemical inhibition of the mitochondrial fission protein Drp1 leads to increased melanin production and elongated mitochondria, whereas downregulation of the mitochondrial fusion protein dynamin-like GTPase 1 suppressed melanogenesis and caused mitochondrial fragmentation. Correspondingly, fragmentation of mitochondria induced by carbonyl cyanide m-chlorophenyl hydrazone (CCCP) treatment also reduced melanogenesis. Additionally, mitochondrial fission triggered the ROS-ERK pathway, leading to the proteasomal degradation of the Microphthalmia-associated transcription factor, thereby accelerating the suppression of melanogenesis, supporting the hypothesis that mitochondrial dynamics have an impact on melanogenesis (Kim et al., 2014).

A recent study examined the impact of mtDNA depletion on melanocyte function and structure using a mtDNA-depleted mice model (Villavicencio et al., 2021). This study has shown that mtDNA-depleted mice exhibit increased pigmentation along with highly dendritic melanocytes, resembling characteristics observed in senile lentigo, an age-related hyperpigmentation disorder. This study highlights the potential value of this mouse model to be used as a tool to investigate how mitochondrial damage contributes to skin pigmentation disorders, potentially paving the way for the development of novel therapeutic approaches targeting pigmentation disorders and premature skin aging (Villavicencio et al., 2021).

Recent studies demonstrate that melanocytes also actively contribute to skin aging through their secretory activity. The secretome of senescent melanocytes was shown to affect surrounding cells such as keratinocytes impairing their proliferation and leading to changes in the skin microenvironment (Victorelli and Passos, 2020; Kang et al., 2021). The appearance and persistence of senescent melanocytes in the skin have been reported to contribute to other age-related changes and pathologies of this tissue such as increased susceptibility to infection, impaired wound healing, and tumorigenesis (Wang and Dreesen, 2018). A recent study revealed that UV-induced senescent melanocytes experience impaired functionality in transporting melanosomes to the dendrites of melanocytes, and this phenomenon is believed to be associated with mitochondrial dysfunction. Moreover, the study unveiled that as melanocytes enter senescence, there is an increase in glycolysis. The inhibition of glycolysis reduces senescence-associated characteristics resulting in an improvement in melanosome transport and distribution. These findings highlight that glucose metabolism plays an essential role in the development of melanocyte senescence (Park et al., 2023).

While melanocytes significantly impact the skin microenvironment through their secretome, their function and metabolism are also regulated by secretory factors such as extracellular vesicles, cytokines, and other soluble factors originating from various cells, including keratinocytes, fibroblasts, and sebocytes (Okazaki et al., 2005; Yoon et al., 2018; Shi et al., 2021; Flori et al., 2022). This process of intercellular communication can lead to the onset of skin pigmentation disorders such as melasma and vitiligo (Bastonini et al., 2019; Flori et al., 2022). Notably, UV-irradiated fibroblasts have been observed to suppress the expression and secretion of stromal cell-derived factor 1 (SDF-1) thereby playing a role in the development of senile lentigines (Yoon et al., 2018). These findings emphasize the complexity of factors driving skin aging and skin pigmentation disorders, highlighting the importance of investigating the mechanisms governing intercellular communication across diverse skin cell types.

The main mitochondrial damages observed in skin melanocytes during aging, along with their resulting consequences for the skin, are displayed in Figure 3.

### 4.3 Mitochondria in fibroblasts homeostasis and aging

Skin or dermal fibroblasts play a vital role in supporting the structure and function of the skin. They are responsible for synthesizing and maintaining the ECM and actively participate in other skin-related processes such as wound healing. Being energy-demanding cells, fibroblasts heavily rely on mitochondria to carry out their crucial functions, including ECM synthesis, secretion of growth factors and cytokines, and cellular signaling. Furthermore, mitochondrial function is critical for sustaining fibroblast viability, promoting proliferation, and facilitating collagen synthesis, all of which are essential for maintaining tissue integrity (Krutmann and Schroeder, 2009; Katsuyama et al., 2022; Yanes and Rainero, 2022).

During aging, fibroblasts aggregate damaged mitochondria and mtDNA deletions leading to structural and functional alterations in the ECM and the induction of inflammation, which in turn, accelerates the formation of skin wrinkles (Krutmann and Schroeder, 2009; Ho and Dreesen, 2021). Fibroblasts derived from aged skin display many of the characteristics associated with aging and mitochondrial damage. These characteristics include impaired mitochondrial respiration as reflected by increased baseline respiration and proton leakage along with reduced ATP-linked respiration in combination with increased mitochondrial mass and ROS production (Rorteau et al., 2022). Interestingly, chronologically aged fibroblasts display a decline in both the expression and activity of mitochondrial Complex 2 (Bowman and Birch-Machin, 2016; Schniertshauer et al., 2018).

Irradiation of fibroblasts can lead to mtDNA mutations. These mutations, in turn, result in mitochondrial dysfunction, oxidative stress, and reduced collagen production which consequently promotes the expression of collagen-degrading proteins like matrix metalloproteases (Schroeder et al., 2008; Sreedhar et al., 2020; Katsuyama et al., 2022). These molecular characteristics are strongly associated with photo-aged skin and wrinkling which provide evidence that mitochondrial dysfunction and degradation of the ECM can contribute to skin aging (Sreedhar et al., 2020). Moreover, the depletion of mtDNA in mice induced by doxycycline treatment leads to inflammatory and wrinkled skin, accompanied by a hyperkeratotic epidermis and hair loss (Singh et al., 2018).

Recent research by Wedel et al. (2023) sheds light on alterations in mitochondrial fission and fusion processes within skin fibroblasts during skin aging. Their study suggests that a reduced rate of mitochondrial fission events or an increased propensity for hyperfusion may contribute to the premature elongation of mitochondria in senescent skin fibroblasts with decreased growth differentiation factor 15 (GDF15) expression (Wedel et al., 2023). These results are in contrast with a study published by Sgarbi and others (Sgarbi et al., 2014) in which they have demonstrated that fibroblasts derived from centenarians skin preserve mitochondrial bioenergetics function by remodeling mitochondrial network towards hyperfusion. These studies shed light on mitochondrial dynamic changes in aged skin fibroblasts, underscoring the significance of further exploration to unravel the complex mechanisms governing mitochondrial dynamics in skin aging.

Recent studies have shed light on mechanisms of intercellular communication elicited by factors secreted by senescent fibroblasts ultimately leading to skin aging. For instance, the relationship between skin pigmentation and components of the SASP secreted by senescent dermal fibroblasts, including SDF-1 and GDF15 has recently been addressed (Yoon et al., 2018; Kim et al., 2020). These factors have been associated with the development of skin pigmentation disorders (Conte et al., 2022). GDF15, specifically, has emerged as a novel marker for aging (Kim et al., 2020) and senescence (Flori et al., 2022). It is activated in response to cellular stress, inflammation, and mitochondrial dysfunction, and is categorized as a stress response cytokine and mitokine (Conte et al., 2022). Accumulated senescent fibroblasts during skin aging exhibit high expression of GDF15 (Kim et al., 2020), and depletion of GDF15 in fibroblasts induces mitochondrial dysfunction, cellular senescence, and age-related skin alterations when incorporated into skin equivalents (Wedel et al., 2023). Furthermore, when UVA-induced senescent fibroblasts with high secretion of GDF15 are co-cultured with melanocytes, the pigmentation process of melanocytes is upregulated, potentially leading to skin pigmentation disorders (Kim et al., 2020). In contrast, the expression and secretion of SDF-1 is suppressed in senescent fibroblasts leading to stimulation of melanogenesis and thereby playing a role in the development of skin pigmentation disorder (Yoon et al., 2018). These recent publications highlight the significance of the secretory behavior of dermal fibroblasts for the environment of the skin and how mechanisms of intra-tissue and intercellular communication ultimately related to mitochondrial function contribute to skin aging.

A distinct form of cellular senescence, arising from mitochondrial dysfunction and distinguished by a unique secretion profile lacking the inflammatory interleukin-1 (IL-1) arm has been identified in human fibroblasts and named mitochondrial dysfunction-associated senescence (MiDAS). In this study, the authors demonstrated that the senescence-associated secretory phenotype of fibroblasts induced by MiDAS stimulates keratinocyte differentiation supporting the hypothesis that the secretome of MiDAS cells induce the rapid accumulation of senescent-like keratinocytes, which show enhanced differentiation and might drive aging phenotypes and age-related pathologies in the skin (Gallage and Gil, 2016; Wiley et al., 2016).

UV exposure leads to decreased barrier function, alterations in skin physiology, and changes in mitochondrial morphology and function in comparison to non-exposed skin (Kimball et al., 2018). It was reported that in sun-exposed skin samples collected from donors of varying ages, mitochondrial damage predominantly occurs in dermal fibroblasts and is accompanied by reduced expression of crucial oxidative phosphorylation genes and subunits of mitochondrial complexes (Oblong et al., 2020). The group conducting this study proposed a hypothesis suggesting that this mitochondrial damage is attributed to decreased mitophagy activity, which could potentially be restored through treatment with nicotinamide (Oblong et al., 2020). Similarly, a study has reported that UV-irradiated fibroblasts exhibit reduced production of collagen type 1 and fibrillin 1, indicating impaired secretion of ECM proteins. This impairment is hypothesized to be linked to mitochondrial quality control, particularly due to low ATP production (Katsuyama et al., 2022). Additionally, it was shown that UVB exposure leads to the downregulation of Mfn proteins and the upregulation of Drp1, disrupting the balance of fusion-fission events and resulting in mitochondrial fragmentation in fibroblasts (Gupta et al., 2022).

The main mitochondrial damages observed in skin fibroblasts during aging, along with their resulting consequences for the skin, are displayed in Figure 3.

### 4.4 Mitochondrial age-related changes of skin immune resident cells

During aging, the progressive decline of immune function and reduction in the immune system’s ability to respond effectively to new and emerging challenges leads to immunosenescence which is characterized by the deregulation of immune responses, impaired healing, and poor tissue restoration and function (Pilkington et al., 2021). The aging process also results in a decline in the number and function of immune cells in the skin. For example, the number of epidermal Langerhans cells, a type of dendritic cell, decreases with age, reducing the capacity to stimulate T cells and initiate an immune response (Russell-Goldman and Murphy, 2020). Additionally, during the process of skin aging naive-to-memory T cell ratios are decreased, effector function is impaired, the receptor repertoire of T cells is restricted, and there is a reduction in antibody diversity. Immunosenescence is often accompanied by chronic, low-grade systemic inflammation that is referred to as inflammaging. This is driven by elevated levels of inflammatory cytokines, such as interleukin-6 (IL-6), interleukin-8 (IL-8), and tumor necrosis factor-α (TNF- α), which are secreted by both innate immune cells and non-immune cells such as dendritic cells (DCs), neutrophils, macrophages, and fibroblasts that become dysfunctional during the aging process (Pilkington et al., 2021). Finally, age-related changes in the skin can directly impact the function of immune cells and contribute to immune dysfunction. For instance, skin aging is associated with a decrease in collagen production and an increase in elastin degradation, leading to wrinkle formation and decreased mechanical strength (Quan and Fisher, 2015; McCabe et al., 2020). These changes may impair immune cell migration, antigen presentation, and tissue repair.

Mitochondria are essential for regulating immune cell activation, differentiation, metabolism, effector functions, and immune survival. Therefore, cells from the innate and adaptive immune system rely heavily on mitochondria to maintain their fate and function (Cervantes-Silva et al., 2021). It is well accepted that individual immune cells have distinct metabolic requirements, which guide their effector function throughout the immune response against different pathogens. In fact, changes in immunometabolism have been highlighted as a major modifier of immune cell function during senescence and aging (Martin et al., 2021). Additionally, given the importance of the mitochondrial metabolism for maintenance of homeostasis of immune cells, it is not surprising that upon infection, pathogens often aim to manipulate these organelles for their benefit and have developed strategies to interfere with the host cell’s mitochondrial remodeling, which ultimately helps the microorganisms to survive (Cervantes-Silva et al., 2021). In this chapter, we will highlight the main metabolic changes happening in the skin immune system during aging, given a special focus on resident populations of immune cells in skin tissue and how their mitochondria and consequently their metabolism are affected during aging. It is important to emphasize that most of the research on age-related changes in mitochondrial metabolism of immune cells is not performed in the skin. This highlights the necessity of new studies focusing on changes in skin immunometabolism during aging.

The skin surface is inhabited by a complex community of microorganisms, including bacteria, fungi, and viruses, recognized as skin microbiota. These microorganisms play a vital role in maintaining skin health by protecting against pathogenic invaders and regulating immune function (Abdallah et al., 2017). Age-related changes in the skin, such as decreased sebum production and alterations in skin pH, can significantly impact the composition and diversity of the skin microbiome. It is also suggested that age-related changes in the skin’s immune system, such as a decreased ability to produce antimicrobial peptides, may contribute to alterations in the skin microbiome. In addition, exposure to environmental factors such as UV radiation and pollution can also affect the skin microbiome, leading to changes in microbial diversity and composition (Abdallah et al., 2017; Guttman-Yassky et al., 2019). Considering the probable bacterial origins of mitochondria, the possibility of microbiota-mitochondria interactions in host cells is conceivable. ROS may serve as a focal point for this crosstalk (Chartoumpekis et al., 2022). Nonetheless, the precise molecular mechanisms of such communication and their alterations during the aging process remain undisclosed, thereby exceeding the remit of this review article. In regards to the skin resident cells, the number of DCs, as well as their ability to recognize and present antigens decreases with age, leading to a decline in immune response. This can contribute to an increased susceptibility to skin infections and cancers in older adults (Wong and Goldstein, 2013). In murine models, aged DCs displayed marked indications of mitochondrial impairments, such as decreased ΔΨ_M_ and ATP turnover and coupling efficiency, reduced baseline OXPHOS, and increased proton leakage and ROS generation. These alterations in mitochondrial bioenergetics cause a depletion of phagocytic and antigen cross-presenting capacity of DCs and consequent alterations in immune response (Chougnet et al., 2015). In humans, there is limited research on the specific changes that occur in mitochondria of DCs during aging. Similar to what is reported in mice, aging leads to alterations in mitochondrial function, including changes in OXPHOS and ROS generation. Studies have shown that aging DCs exhibit decreased antigen presentation capacity and reduced ability to activate T cells (Wong and Goldstein, 2013). Mitochondrial dysfunction in aged DCs may contribute to these changes, as DCs rely on mitochondrial metabolism to meet their higher energy demands for antigen processing and presentation. Additionally, impaired mitochondrial function may make DCs more susceptible to stress and apoptosis, impacting the numbers of functional DCs in aged tissues (Chougnet et al., 2015; Quinn et al., 2018). Overall, further research is necessary to understand the precise mechanisms and consequences of mitochondrial changes in aged DCs.

During aging of the skin, the number and function of T cells decrease, impairing the immune response. This decline in T cells is mainly due to thymic involution, a process in which the thymus gland shrinks and loses its ability to produce new T cells. Additionally, the remaining T cells become less efficient in recognizing and responding to antigens, leading to a weakened immune system. This decline in T cell function contributes to the development of age-related skin diseases and impaired wound healing (Koguchi-Yoshioka et al., 2021). Several studies have identified age-related changes in mitochondrial function, including decreased ΔΨ_M_, ROS production, and altered expression of mitochondrial genes in T cells. For instance, it is well accepted that mitochondrial dysfunction alters cellular metabolism, increases oxidative stress, and is involved in dysregulated signaling and function of cluster of differentiation (CD)4^+^ T lymphocytes in the elderly (Headley et al., 2021). A recent study has investigated whether aging-associated mitochondrial dysfunction could be abrogated by transferring young mitochondria into CD4^+^ T cells from old mice and whether such transfer changed CD4^+^ T cell function. The results of this study demonstrated that mitochondrial transfer improved the redox status and function of CD4^+^ T cells from old mice. These findings support the notion that mitochondria can serve as targets of therapeutic intervention in aging (Headley et al., 2021). Another study has demonstrated that activated T cells isolated from old mice exhibit increased C14/C16 ceramide accumulation in mitochondria, triggered by ceramide synthase 6, which in turn leads to mitophagy dysfunction and accumulation of damaged mitochondria highlighting a potential therapeutic strategy for reversing this process by targeting ceramide-dependent mitophagy (Vaena et al., 2021). However, to the best of our knowledge, there are no studies evaluating specifically mitochondrial function in T-lymphocytes during skin aging, and more research is needed to fully understand the mechanisms and clinical implications of mitochondrial changes in skin T-cells during aging.

The main mitochondrial damages observed in skin immune cells during aging, along with their resulting consequences for the skin, are displayed in Figure 3.

### 4.5 Mitochondrial aging-related changes of sebaceous glands and sebocytes

Sebaceous glands are exocrine glands that are responsible for the secretion of lipid-rich sebum, a critical component of epidermal barrier function, thermoregulation, skin elasticity, and skin/hair microbiome management (Ahmed et al., 2022). Sebaceous glands are typically found in association with hair follicles, and their structure consists of a single duct that opens into the hair follicle, with a cluster of secretory cells at the base of the duct. The glandular cells are primarily composed of sebocytes, which are specialized cells that produce and accumulate lipids and other substances that make up the sebum (Lovászi et al., 2017; Wikramanayake et al., 2022). Given the high metabolic activity of sebocytes dedicated to the synthesis of lipids of the sebum, the cytoplasm of these cells is rich in highly active mitochondria (Schneider and Paus, 2010).

As we age, our skin undergoes a number of changes that affect the function and appearance of sebaceous glands and sebocytes. These changes are driven by several factors, including hormonal shifts, oxidative stress, and the accumulation of cellular damage over time (Woo et al., 2019). Disorders of the sebaceous gland are of particular importance in the pathogenesis of various diseases associated with human aging as well as in the process of skin aging itself. Alterations in the morphology and activity of sebocytes directly affect skin homeostasis and have a great impact on the process of skin aging. During skin aging, the number of sebocytes is not changed, however, the size of these cells is reduced leading to a decreased secretory output of the sebaceous glands (Makrantonaki et al., 2006). This lowers the level of surface lipids and causes skin xerosis, a common condition found in elderly persons. A recent study has demonstrated that myelin protein zero-like 3, an Ig-like v-type mitochondrially localized nucleus-encoded protein that regulates lipid metabolism, energy expenditure, and the differentiation and function of epidermal keratinocytes, acts as a negative regulator of sebaceous gland size and proliferation (Wikramanayake et al., 2022). Although the relationship between myelin protein zero-like 3 and age-related changes in sebaceous glands has not been established, this mitochondrial protein may participate in regulating physiological parameters of sebocytes during aging. Additionally, a recent study has reported that factors secreted by stressed sebocytes actively participate in the pathogenesis of melasma (Flori et al., 2022) highlighting the importance of sebaceous glands for skin homeostasis. Although mitochondrial activity plays an important role in sebocytes’ physiology, very few studies have addressed how mitochondrial dysfunction occurs during the aging process.

The main mitochondrial damages observed in skin sebocytes during aging, along with their resulting consequences for the skin, are displayed in Figure 3.

### 4.6 Mitochondrial aging-related changes of cutaneous adipose tissue

During aging the adipose tissue undergoes significant changes in mass and distribution, characterized by an increase in visceral fat and a decrease in subcutaneous fat. Visceral adipose tissue is associated with detrimental metabolic effects, while subcutaneous adipose tissue is considered metabolically beneficial. Age-related peripheral fat loss may be attributed to defects in adipogenesis and heightened inflammation in subcutaneous fat. Mitochondrial dysfunction in adipose tissue during aging is linked to cellular senescence, chronic inflammation, and decline in stem cell activity, as well as obesity and insulin resistance (Woo et al., 2019; Ou et al., 2022). Senescent adipose progenitor cells display poor differentiation capacity associated with high expression levels of p53 and p16^INK4a^, reduced insulin-dependent glucose transport, and hypertrophic expansion of adipocytes (Krstic et al., 2018; de Lange et al., 2023).

The main mitochondrial damages observed in skin adipocytes during aging, along with their resulting consequences for the skin, are displayed in Figure 3.

## 5 Future perspectives

Recent studies have laid the foundation for understanding the cell type-specific nuances in mitochondrial dynamics and function within the skin. One emerging direction of research is the exploration of mitochondrial interactions among different skin cell types and their implications for tissue physiology. Investigating the crosstalk between keratinocytes, melanocytes, and dermal fibroblasts is anticipated to shed light on how mitochondrial dynamics collectively contribute to skin homeostasis and responses to external stressors.

The importance of mitochondria in human health is evidenced by the consideration of artificial mitochondrial transplant as a possible therapeutic approach to treat damage of several tissues, including the skin (Balcázar et al., 2020). Furthermore, a number of therapeutic options, including the topical application of natural substances and antioxidants targeting mitochondria, are under clinical consideration for the maintenance of functional mitochondria, demonstrating anti-aging effects (Muzammil et al., 2021).

Yet, several important questions remain to be answered. Firstly, how do distinct mitochondrial dynamics in different skin cell types harmonize to maintain overall skin health? Secondly, what role does mitochondrial trafficking play in cellular communication within the skin microenvironment? Additionally, can we harness the knowledge of cell type-specific mitochondrial dynamics to develop targeted interventions for skin conditions, such as aging, inflammation, or UV damage? Furthermore, the identification of key regulators governing mitochondrial dynamics in specific cell types and their potential as therapeutic targets holds great promise. Addressing these questions and delving into the intricacies of mitochondrial behavior across different skin cell types is the next frontier in advancing our understanding of skin biology and paving the way for innovative approaches in dermatology.

## 6 Conclusion

Mitochondria act as dynamic hubs, orchestrating energy production, metabolism, and cellular signaling pathways critical for keratinocytes, fibroblasts, melanocytes, and immune cells. The integrity of mitochondrial function is indispensable for sustaining the specialized functions of each cell type, from keratinocyte differentiation to fibroblast collagen production, melanin synthesis, and immune surveillance. However, as cells age, mitochondrial damage accumulates, impairing their function and triggering a cascade of events that contribute to the multifaceted process of skin aging. This includes disruptions in energy production, increased oxidative stress, and altered cellular signaling, collectively culminating in diminished skin health and the visible signs of aging. Understanding the intricacies of mitochondrial function and dysfunction in these diverse skin cell populations not only sheds light on the molecular mechanisms underlying skin aging but also paves the way for innovative therapeutic strategies targeting mitochondrial health to promote more youthful and resilient skin.

## Figures and Tables

**Table:** 

**ATP**	adenosine triphosphate
**Bcl-2**	C-cell lymphoma 2
**CCCP**	carbonyl cyanide m-chlorophenyl hydrazone
**CD**	cluster of differentiation
**CoQ**	Coenzyme Q
**DCs**	dendritic cells
**DEJ**	dermoepidermal junction
**Drp1**	dynamin-related protein 1
**ECM**	extracellular matrix
**ERK**	extracellular-signal-regulated kinase
**GDF15**	growth differentiation factor 15
**GTP**	Guanosintriphosphat
**IL**	interleukin
**MiDAS**	mitochondrial dysfunction-associated senescence
**Mfn**	mitofusin
**MSCs**	mesenchymal stem cells
**mtDNA**	mitochondrial deoxyribonucleic acid
**NAD+**	nicotinamide adenine dinucleotide
**NAM**	nicotinamide
**NIX/BNIP3L**	BCL2/adenovirus E1B 19 kDa protein-interacting protein 3-like
**NLRP3**	NACHT, LRR and PYD domains-containing protein 3
**OXPHOS**	oxidative phosphorylation
**ROS**	reactive oxygen species
**SA β-Gal**	senescence-associated β-Galactosidase
**SASP**	senescence-associated secretory phenotype
**SDF1**	stromal cell-derived factor 1
**TCA**	tricarboxylic acid
**TFAM**	mitochondrial transcription factor A
**TNF- α**	tumor necrosis factor-α
**UV**	ultraviolet
**ΔΨM**	mitochondrial membrane potential
